# Evaluation of Root Canal Configuration of Maxillary and Mandibular First Molar by CBCT: A Retrospective Cross-Sectional Study

**DOI:** 10.3390/diagnostics12092121

**Published:** 2022-08-31

**Authors:** Rakan Rafdan Alhujhuj, Rizwan Jouhar, Muhammad Adeel Ahmed, Abdullatif Abdulrahman Almujhim, Mohammed Tariq Albutayh, Necdet Adanir

**Affiliations:** Department of Restorative Dental Sciences, College of Dentistry, King Faisal University, Al-Ahsa 31982, Saudi Arabia

**Keywords:** cone-beam computed tomography, endodontics, maxillary first molar, mandibular first molar, root canal configuration, Vertucci’s classification

## Abstract

Adequate knowledge of root canal morphology along with its probable variations is imperative to acquire successful endodontic treatment. This retrospective cross-sectional study aimed to investigate the root canal configuration of maxillary and mandibular first molar using Cone-Beam Computed Tomography (CBCT) among patients in Al-Ahsa region, Saudi Arabia. A total of 377 samples were included, out of which 123 CBCT (I-CAT Vision QTM) scans with intact all first permanent molars were selected in this study and scanned in sagittal, axial, and coronal views by using BlueSkyPlan software. The number of canals in each root and their configuration according to the Vertucci classification system was evaluated. Statistical analysis was analyzed using SPSS version 21 (IBM). Chi-square test was applied to evaluate the association of root canal morphology and mandibular and maxillary first molars with respect to gender. Out of 123 CBCT scans, 59 (48.0%) were males and 64 (52.0%) were females; the mean age was 26.95 ± 10.65 years. The mesiobuccal root of bilateral maxillary first molar had Type-I (87.0%) of Vertucci classification followed by Type-IV (9.8%). Additionally, all mesiobuccal roots (100%) of the left mandibular first molar had Type-I of Vertucci’s classification. A significant association has been observed between gender and a number of canals in bilateral maxillary first molars. Females showed a significantly higher prevalence of three-root canal configuration in maxillary first molars of both sides compared four canals found most commonly in males (*p* = 0.004). This study concluded that the majority of maxillary and mandibular permanent first molars had three roots and three canals with Type-I Vertucci’s classification in patients belonging to the Al-Ahsa region of the Saudi Arabia. It was also proved that gender is significantly associated with the number of canals in a bilateral maxillary molar.

## 1. Introduction

Knowledge of root canal morphology is key to practicing successful endodontic treatment. The occurrence of anatomical variations in the root canal system has always been a leading endodontic challenge to dental practitioners [1].

Endodontic treatment includes cleaning and shaping of root canals, elimination of inflamed or necrotic pulp tissues and subsequently obturation of prepared canals [2]. Failure of endodontic treatment has various reasons, one of which is undetected or missed canals due to complex morphology [3]. Untreated root canals act as reservoirs for bacterial growth which eventually leads to treatment failure [4,5,6].

Vertucci [7] has categorized root canal anatomical patterns of permanent teeth into eight types. Hasan et al. [8] reported that unsuccessful endodontic treatment of maxillary teeth was associated with inappropriate detection and treatment of all canals, particularly the second mesiobuccal canal (MB2). Consequently, dental practitioners must be familiar with probable structural deviation. It has been predicted by some researchers who considered the morphology of maxillary first molar that most cases reported a higher frequency of a second mesiobuccal root (MBR) canal [9]. Similarly, Abd-Latib et al. [10] conducted a study in Malaysia, observing the high prevalence of MB2 canal in maxillary first molar specifically in males. Furthermore, another study reported the prevalence of MB2 canal in 55.8% cases of studied maxillary molars [11]. Likewise, AL-Alawi et al. reported a 4.9% occurrence of radix molars after extraction amongst Malaysians, however radix entomolaris (4.3%) was more prevalent as compared to radix paramolaris (0.3%) [12]. Many researchers have evaluated the morphology of mandibular first molars root canal with respect to the genders and race, where conventional two-dimensional (2D) periapical radiograph and demineralization-staining techniques were used [13,14].

Cone-beam computed tomography (CBCT) is used as a dental diagnostic tool [15,16]. It presents high-quality three-dimensional information and has been shown to be a better technique for pretreatment evaluation of root canal morphology [17]. CBCT is one of the advanced imaging modalities that is capable of overwhelming the superimposition of adjacent structures, and its three-dimensional property makes it superior to conventional periapical radiography [18]. This radiographic modality permits investigation to be done without devastation of tissues and allows normal functioning of natural teeth in the oral cavity.

Studies among the Saudi Arabian sub-population by Al-Fouzan et al. [19] and Alrahabi et al. [20] investigated the morphology of the permanent maxillary first molars by using CBCT (in vitro) and conventional radiographs (in vivo). They showed prevalence of two canals in the MBR estimated as 70.6% in vitro and ranging from 23.3% to 51.3% in vivo. Many researchers have established the superior effect of high-quality three-dimensional images by CBCT over conventional radiographic techniques, in order to study the morphology of root canal system [21,22,23]. CBCT is believed to be an effective adjunctive diagnostic tool that assists in dealing with management, diagnosis, and follow-up [24]. It reflects the accurate similar results that can be obtained by using modified staining techniques and tooth sectioning to identify the variation in root canal morphology [25,26].

Al-Ahsa is in the southeastern part of the Kingdom of Saudi Arabia, where it occupies the southern part of the Eastern Province. It covers a large area of about 530 thousand square kilometers, representing 68% of the Eastern Region and 24% of the Kingdom. The population of Al-Ahsa is over one million. Al-Ahsa is an agricultural oasis embodied by the nature of the place and the abundance of water. There are more than two million palm trees in Al-Ahsa; these trees produce the best types of dates in the world.

Comprehensive information of the most common root and root canal morphologies with their variations is imperative to support dental practitioners in identifying irregularities during root canal therapy to increase successful outcome. To the best of our knowledge, there is no study conducted to explore the root canal morphology of maxillary and mandibular first molars in the Al-Ahsa region of Saudi Arabia. The aim of the current study was to investigate the root canal configuration of maxillary and mandibular first molar using Cone-Beam Computed Tomography (CBCT) in patients belonging to the Al-Ahsa region of Saudi Arabia. 

## 2. Materials and Methods

This retrospective cross-sectional study was conducted at Dental Complex of King Faisal University after obtaining ethical approval from the Research Ethics Committee of Deanship of Scientific Research at King Faisal University (KFU-REC/2020-10-21). Data were collected from digitized CBCT images from October 2020 to September 2021. A total of 377 CBCT scans samples were retrieved from the database by using the convenience sampling technique, out of which 123 CBCT scans fulfilled the inclusion criteria; these were all intact first permanent molars of both genders, with mature apex, and aged from 12–58 years. A total of 254 CBCT scans were excluded for various reasons such as permanent molars with root canal treatment, periapical infections, severe calcification or resorption, inaccurate CBCT scans, compromised anatomy caused by physiological or pathological processes, full-coverage restorations, or metallic restorations.

Permission was obtained from the director of the dental complex to have patients’ demographic details such as gender, age, and CBCT scans to evaluate the number of roots and root canal morphology of maxillary and mandibular first molars. 

**Figure 1:**
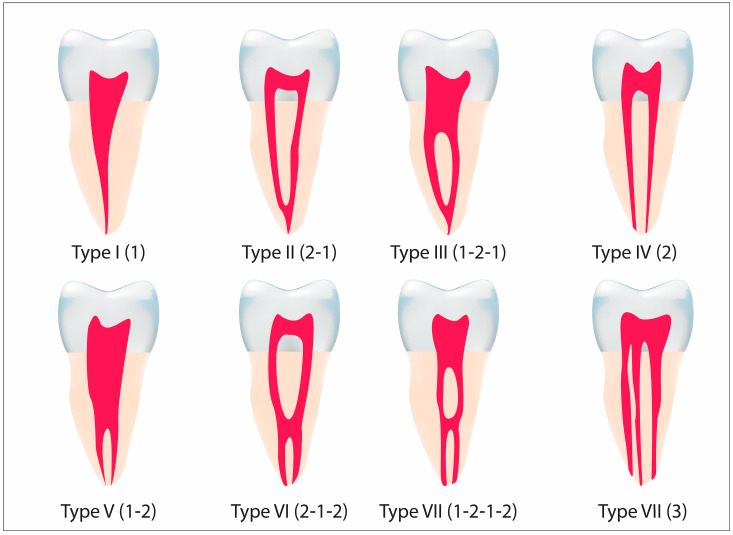
Schematic diagrams of Vertucci’s Classification [27].

Root canal morphology was classified according to Vertucci’s classification system [27] that categorized the canal configurations into eight types as shown in Figure 1.

CBCT scans were captured with I-CAT Vision QTM (Imaging Sciences International, Hatfield, PA, USA. Version 1.9.3.14) 360-degree rotation scans with the field of view 130 with a voxel size of 0.25 mm at 0.250–0.400 Res, 120 kV, and 5 mA with exposure time 2–7 s. Inspection of images was done on a screen with an optimal view. Image brightness/contrast and magnification were adjusted to avoid mistakes and optimize visualization and interpretation. Gathering, interpretation, and recording of CBCT images were carried out by the principal investigator. BlueSkyPlan (Version 4.7.55, GmbH, Langenhagen, Germany) software was used to identify the number of roots and root canal configurations in sagittal, axial, and coronal views while following the checklist of Martins et al. [28].

### Statistical Analysis 

The collected data were analyzed using SPSS Version 21; IBM, Chicago, IL, USA. The number of roots, the number of canals and the configurations of all permanent first molars were documented as frequencies and percentages. Chi-square test was applied to evaluate the association of root canal morphology of mandibular and maxillary first molars with respect to gender. *p*-value < 0.05 was considered as statistical significant.

## 3. Results

A total of 123 CBCT images of the Saudi patients were assessed, out of which 59 (48.0%) were males and 64 (52.0%) were females; the mean age was 26.95 ± 10.65 years. All the patients 123 (100.0%) were Saudi nationals, as shown in Table 1.

Morphology of right maxillary first molar (FDI tooth # 16) revealed that three roots were prevalent in 111 (90.2%) cases while 12 (9.8%) cases had four roots. Three canals were prevalent in 97 (78.9%) cases while 26 (21.1%) cases had four canals. The majority of mesiobuccal root of right maxillary first molar had Type-I 107 (87.0%) of Vertucci classification followed by Type-IV 12 (9.8%), while only 4 (3.3%) patients had Type-II. Almost all palatal root 122 (99.2%) was Type-I and only 1 (0.8%) patient had additional Mesiopalatal canal MP (I). Morphology of left maxillary first molar (FDI tooth # 26) revealed that 111 (90.2%) cases had three roots while 12 (9.8%) cases had four roots. Three canals were prevalent in 94 (76.4%) cases while 29 (23.6%) cases had four canals. Most of the mesiobuccal root of left maxillary first molar was Type-I 104 (84.6%) followed by Type-IV 12 (9.8%), while only 7 (5.7%) patients had Type-II. No difference was found in palatal root finding of left maxillary and right maxillary molars. Distobuccal canal of all the bilateral maxillary first molars was Type-I. As far as other configuration of bilateral maxillary molars is concerned, it was observed that 10 (8.1%) had second mesiobuccal canal MB2 (I) and 1 (0.8%) had Disto-palatal DP (I), as shown in Table 2.

Morphology of left mandibular first molar (FDI tooth # 36) revealed that 105 (85.4%) cases had three roots followed by four roots in 15 (12.2%) cases. On the other hand, most of the patients 90 (73.2%) had three canals while 31 (25.2%) had four canals. All the patients 123 (100.0%) of mesiobuccal root of left mandibular first molar had Type-I of Vertucci’s classification. Mesiolingual canal of 122 (99.2%) patients had Type-I followed by Type-II in 1 (0.8%) case. Morphology of Distal canal showed that 91 (74.0%) were Type-I, 9 (7.3%) were Type-II, 2 (1.6%) were Type-III, 3 (2.4%) each in Type-IV and V and 15 (12.2%) cases showed additional Distobuccal (I) canal. As far as other configuration is concerned, it was observed that only 15 (12.2%) had Distolingual (I) canal. Morphology of right mandibular first molar (FDI tooth # 16) revealed that 105 (85.4%) cases had three roots followed by four roots in 16 (13.0%) cases and two roots present in 2 (1.6%) cases. On the other hand, most of the patients 91 (74.0%) had three canals while 31 (25.2%) had four canals followed by two canals in 1 (0.8%) case. All the patients 123 (100.0%) of mesiobuccal root of right mandibular first molar was Type-I of Vertucci’s classification. Mesiolingual canal of 122 (99.2%) patients was Type-I followed by Type-II in 1 (0.8%) case. Morphology of the Distal canal showed that 91 (74.0%) were Type-I, 7 (5.7%) were Type-II, 2 (1.6%) were Type-III, 5 (4.1%) were Type-IV and 2 (1.6%) were Type-V and 16 (13.0%) cases showed additional distobuccal (I) canal. As far as other configuration is concerned, it was found that only 16 (13.0%) had Distolingual (I), as shown in Table 3. 

A significant association was observed between gender and number of canals in maxillary right first molar. Females showed significantly higher prevalence of three-root canal configuration in maxillary first molars of the right side compared to males, while a majority of males had four canals (*p* = 0.004). In contrast, an insignificant association was found between gender and number of roots and other configuration of maxillary right first molar (*p* > 0.05). Concerning maxillary left first molar, a significant association was observed between gender and number of canals. Most females had three canals compared to males (*p* = 0.001). Additionally, an insignificant association was found between gender and number of roots and other configurations of maxillary left first molar (*p* > 0.05), as shown in Table 4.

On the other hand, there was an insignificant association observed between gender and number of canals, number of roots, and other configurations of mandibular left and right first molars (*p* > 0.05), as shown in Table 5.

**Figure 2:**
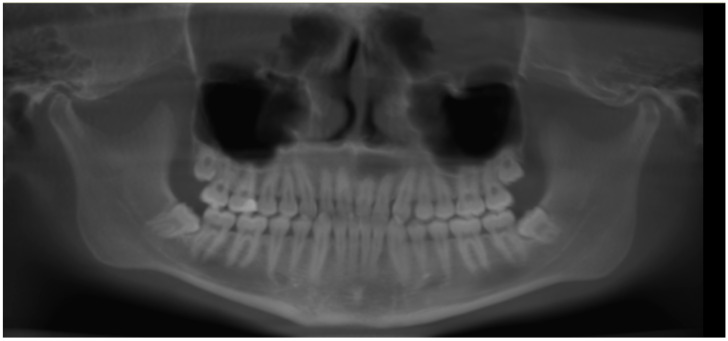
Panoramic view showing intact all permanent first molars.

**Figure 3:**
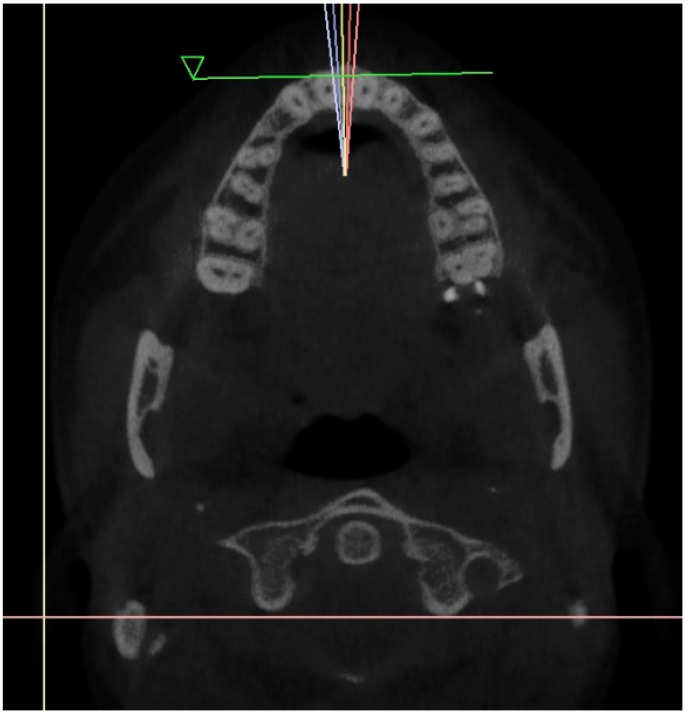
Axial view of maxillary arch showing number of roots and canals in maxillay molars.

**Figure 4:**
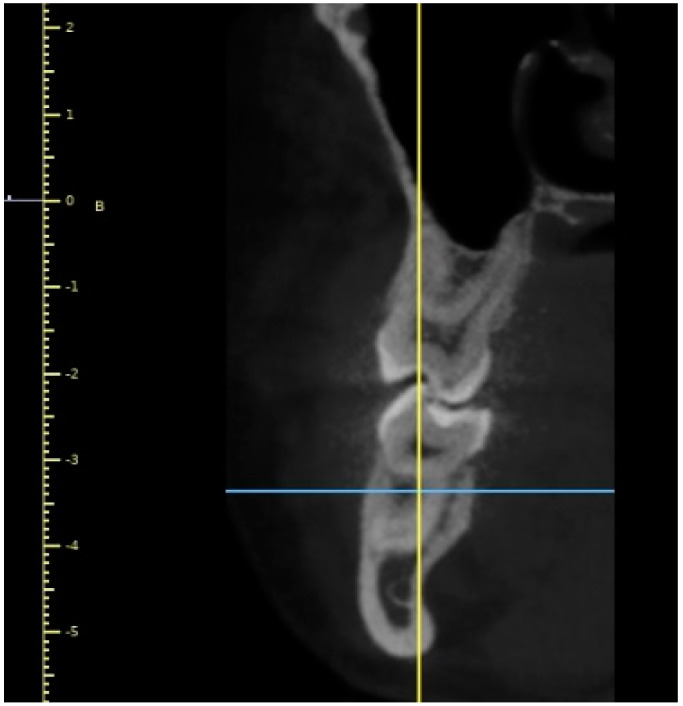
Coronal view of maxillary right molar (#16) showing Vertucci’s Type-I canal configuration.

Initially, a panoramic image of patients was used for a general dental evaluation and select cases according to inclusion criteria as shown in Figure 2. An axial view of the maxillary arch was used to evaluate the number of roots and canals in maxillary molars as shown in Figure 3. Subsequently, the coronal view was used to evaluate the type of Vertucci’s classification in the palatal root of maxillary first molar as shown in Figure 4.

## 4. Discussion

It is imperative to ponder the complicated three-dimensional root canal morphology and probable variations to have successful endodontic treatment. A detailed theoretical interpretation, and hence an understanding of root canal morphology, can significantly diminish the problematic tasks encountered in access cavity preparation, cleaning and shaping, obturation, and final restoration of the root canal system [29].

The clinical efficiency of endodontic treatment procedures is dependent on a comprehensive knowledge of root structure and variance probability in root canal configuration, as undetected root canals lead to a treatment failure [30]. The present study demonstrated the root structure and root canal configuration of maxillary and mandibular first molars by using CBCT and its association with gender in the Saudi population.

Recently in clinical dental practice, the application of CBCT has gained admiration. With its non-invasive technique, CBCT supports dental practitioners in the diagnosis of endodontic problems along with a correct management plan. CBCT has been demonstrated as dependable and auspicious in identifying root canal structures in comparison with typical visual examination through physical tooth sectioning [31]. In 2015, the *American Association of Endodontists and American Academy of Oral and Maxillofacial Radiology Joint Position Statement* proposed that dental practitioners were recommended to apply CBCT merely while conventional dental radiography was unable to satisfactorily capture the images [32].

Interestingly, the accuracy of identifying additional canals is dependent on the voxel measurement and contrast resolution application. The present study scanned the maxillary and mandibular first molar teeth with voxel size 0.25 mm at 0.250–0.400 Res which gives an optimal view. This was endorsed by Bauman et al., who signified the resolution in the CBCT system that is ≤0.2 mm voxel sizes are optimum for the recognition of MBR canals [33]. Similarly, another research supported the CBCT system wherein all CBCT machines were fixed at 0.3 mm voxel size (14-bit grayscale) which gave optimal results [34]. 

This voxel measurement offered better diagnostic management and minimized the radiation dose; additionally, it looks suspicious that it had a great influence on the reliability of extra canal detection [33,35].

The present study showed that maxillary and mandibular first molars had three separate root canals, which were consistent with another research project wherein most of the first molars had three separate root canals (94%) [34]. These findings showed similarities with the former CBCT results in Saudi [19] and Brazilian populations [36]. 

The second mesiobuccal canal (MB2) is one of the most commonly undetected canals in maxillary molars that escapes the pulp chamber at a critical mesial inclination, and consequently bends distally making the task difficult for dental practitioners [37]. The present study showed that the prevalence of an MB2 in maxillary first molars was 10 (8.1%), which was not in accordance with other CBCT research wherein the prevalence of an MB2 was reported at 36.3% [38], <50% [39] and 73.8% [40]. Furthermore, our result is contrary to a Micro-CT based study [41] which showed that MB2 was present in 80% of the first maxillary molar. These differences in the results may be accredited to dissimilar sample sizes, ethnic groups, and approach variances. Previous studies showing the presence of MB2 canal in maxillary first molar in different populations is summarized in Table 6.

**Table 6:** Studies show in the presence of MB2 canal in maxillary first molar in different population.

Studies	Year	Population	CBCT Scans	Number of Canals in the Maxillary First Molar
Kim et al. [9]	2012	Korean	814	Additional canals were found in 63.59% of the mesiobuccal (MB) roots
Silva et al. [36]	2014	Brazilian	314	The prevalence of MB2 canal was 44.4%
Martin et al. [40]	2018	Indian	250	MB2 proportion in USA was 64.8%.
Al Mheiri et al. [42]	2020	Emirati	261	MB2 canal was found in 80.1% of all examined samples.
Martin et al. [40]	2018	Australian	250	50.8% samples had MB2 canal
Martin et al. [43]	2017	Caucasian	542	The mesiobuccal root of the maxillary first molars had two root canals in 71%
Heibert et al. [44]	2017	American	100	A review of CBCT volumes found the presence of an MB2 canal 69% of the time.
ABARCA et al. [45]	2015	Chilean	802	The presence of the MB2 canal in maxillary first was 32.5%.
Jing et al. [46]	2014	Chinese	630	30.9% maxillary molars had the additional canal in the mesio-buccal roots.
Naseri et al. [47]	2016	Irani	149	Additional canal (MB2) was detected in 86.6% of mesiobuccal roots

Besides MB2, the prevalence of second palatal canal in maxillary molars is rare. However, the present study identified the prevalence of second palatal canal 1 (0.8%) that was consistent with other research that observed the prevalence of a second palatal canal at 0.9% in maxillary first molars [38], and also in agreement with the further research by Ratanajirasut et al. [48] and Martins et al. [49] who revealed the existence of two palatal canals 1.7% of maxillary first molars in the Caucasian group.

Similarly, the present study showed that mandibular first molars of both sides had Type-I configuration, these findings showing inconsistency with findings reported in the studies by Pan et al. [38], Miloglu et al. [50], and Kim et al. [46] wherein most of the mandibular first molars had Type-IV configuration in the mesial root. These variations may be due to differences in sampling populations such as Malaysian, Turkish and Korean populations, respectively, selected in those research projects. 

As far as the distal root is concerned, the present study revealed the prevalence of two distal canals in the distal root of mandibular first molars wherein the second distal canal in the distolingual position was found to be 15 (12.2%) of Type I configuration. These findings partly corroborated with the study by Martins et al. [49] that revealed a 19.5% prevalence rate for a second distal canal in mandibular first molars. Conversely, one of the research projects conducted on the Sudanese population by Ahmed et al. [51] found a higher prevalence of 59% for two canals in distal roots of mandibular first molars. These variations may be due to differences in the sampling population.

Regarding the association of mesiobuccal root of maxillary molar teeth with gender, the present study revealed that mesiobuccal root had Vertucci Type-I that was more common in females, though there was a statistically insignificant difference in comparison with males (*p* > 0.05). These results corresponded strongly with the study conducted in North America by Guo et al. [47], who assessed maxillary first molars and established that Vertucci classification of Type-I was more commonly found in females than males even though there was a statistically insignificant difference between them. Additionally, further research by Betancourt et al. [52] in Chile and Jing et al. [53] in China, also reported that females had a significantly increased prevalence of single-root canal in the maxillary first molar. Remarkably, the present study contradicted the two studies by Altunsoy et al. [54] that were performed on a Turkish population and Naseri et al. [55] on an Iranian population, which supported that males had a higher prevalence of Vertucci Type-I configurations in mesial buccal roots of bilateral maxillary first molars although insignificant association was observed.

The present study concluded that the majority of cases in mandibular first permanent molars of both sides had 3 roots and three canals with insignificant association with gender (*p* > 0.05). These findings were in line with Deng et al.’s [56] research in the population of East Coast Malaysia.

The results of the present study must be seen under certain limitations. The data in the present study were acquired from a single center that maybe limited its methodology. Secondly, after following the exclusion criteria we ended up with 123 patients’ CBCT images with a mean age of 26.95 ± 10.65 years, which is unlikely to represent the all-age group. Therefore, multicenter studies with wider age brackets are recommended for better representation of the population in the future. Additionally, to yield stronger conclusions it is preferable to perform this anatomical study over an extensive geographical range with racial variables. The present study evaluated the root canal morphology of permanent first molars in the Al Ahsa region population that used a voxel size of 0.25 mm and limits the study outcomes. Moreover, visualization of the morphology of root canals can be improved by using a high-resolution imaging modality such as Micro-CT [57,58]. CBCT is an operational, high-precision diagnostic tool for precise anatomical details. Nonetheless, high cost and radiation dose limit its clinical application. Consequently, better outcomes of endodontic treatment can be greatly developed by the acquaintance of clinicians with different root and canal morphological configurations.

## 5. Conclusions

This study concluded that the majority of maxillary and mandibular first molars had three roots and three canals with Type-I Vertucci’s classification. In addition, gender was significantly associated with the number of canals in the maxillary molar, with females showing a greater tendency to have three canals than males. However, multicenter studies with a wider age bracket are recommended for better representation of the population in the future.

## Figures and Tables

**Table 1:** Demographic characteristics of participants (n = 123).

Variables		Mean ± SD n (%)
Age (years)		26.95 ± 10.65
Gender	Male	59 (48.0%)
	Female	64 (52.0%)
Nationality	Saudi	123 (100.0%)

**Table 2:** Distribution of the morphology of the root canal according to Vertucci classification in maxillary first molars.

Variables			n (%)
Tooth # 16 (first molar right side)	mesio-buccal (MB)	I	107 (87.0%)
		II	4 (3.3%)
		III	0 (0%)
		IV	12 (9.8%)
	distobuccal (DB)	I	123 (100.0%)
	Palatal	I	122 (99.2%)
		Mesiopalatal MP (I)	1 (0.8%)
	other configuration	Second mesiobuccal canal MB2 (I)	10 (8.1%)
		Disto-palatal DP (I)	1 (0.8%)
		No	112 (91.1%)
	Number of Roots	3	111 (90.2%)
		4	12 (9.8%)
	Number of Canals	3	97 (78.9%)
		4	26 (21.1%)
Tooth # 26 (first molar left side)	mesio-buccal (MB)	I	104 (84.6%)
		II	7 (5.7%)
		IV	12 (9.8%)
	Disto buccal (DB)	I	123 (100.0%)
	Palatal	I	122 (99.2%)
		Mesiopalatal (I)	1 (0.8%)
	other configuration	Second mesiobuccal canal.MB2 (I)	10 (8.1%)
		Disto-palatal DP (I)	1 (0.8%)
		No	112 (91.1%)
	Number of Roots	3	111 (90.2%)
		4	12 (9.8%)
	Number of Canals	3	94 (76.4%)
		4	29 (23.6%)

**Table 3:** Distribution of the morphology of the root canal according to Vertucci classification in mandibular first molars.

Variable			n (%)
Tooth # 36 (first molar left side)	mesio-buccal (MB)	I	123 (100.0%)
	Mesiolingual (ML)	I	122 (99.2%)
		II	1 (0.8%)
	Distal	I	91 (74.0%)
		II	9 (7.3%)
		III	2 (1.6%)
		IV	3 (2.4%)
		V	3 (2.4%)
		Distobuccal (I)	15 (12.2%)
	other configuration	Distolingual (I)	15 (12.2%)
		No	108 (87.8%)
	Number of Roots	1	1 (0.8%)
		2	2 (1.6%)
		3	105 (85.4%)
		4	15 (12.2%)
	Number of Canals	1	1 (0.8%)
		2	1 (0.8%)
		3	90 (73.2%)
		4	31 (25.2%)
Tooth # 46 (first molar right side)	mesio-buccal (MB)	I	123 (100.0%)
	Mesiolingual (ML)	I	122 (99.2%)
		II	1 (0.8%)
	Distal	I	91 (74.0%)
		II	7 (5.7%)
		III	2 (1.6%)
		IV	5 (4.1%)
		V	2 (1.6%)
		Distobuccal (I)	16 (13.0%)
	other configuration	Distolingual (I)	16 (13.0%)
		No	107 (87.0%)
	Number of Roots	2	2 (1.6%)
		3	105 (85.4%)
		4	16 (13.0%)
	Number of Canals	2	1 (0.8%)
		3	91 (74.0%)
		4	31 (25.2%)

**Table 4:** Association of prevalence of the morphology of the root canal types in maxillary first molars with respect to gender.

Variable			Male Mean ± S.D n (%)	Female Mean ± S.D n (%)	*p*-Value
Age (years)			27.57 ± 11.32	26.37 ± 10.05	0.534
Nationality		Saudi	59 (100.0%)	64 (100.0%)	-
Tooth # 16 (first molar right side)	mesio-buccal (MB)	I	48 (81.4%)	59 (92.2$)	0.195
		II	3 (5.1%)	1 (1.6%)	
		IV	8 (13.6%)	4 (6.3%)	
	distobuccal (DB)	I	59 (100.0%)	64 (100.0%)	-
	Palatal	I	58 (98.3%)	64 (100.0%)	0.296
		Mesiopalatal MP (I)	1 (1.7%)	0 (0.0%)	
	other configuration	Second mesiobuccal canal. MB2 (I)	7 (11.9%)	3 (4.7%)	0.193
		Disto-palatal DP (I)	1 (1.7%)	0 (0.0%)	
		No	51 (86.4%)	61 (95.3%)	
	Number of Roots	3	51 (86.4%)	60 (93.8%)	0.172
		4	8 (13.6%)	4 (6.3%)	
	Number of Canals	3	40 (67.8%)	57 (89.1%)	0.004
		4	19 (32.2%)	7 (10.9%)	
Tooth # 26 (first molar left side)	mesio-buccal (MB)	I	45 (76.3%)	59 (92.2%)	0.050
		II	5 (8.5%)	2 (3.1%)	
		IV	9 (15.3%)	3 (4.7%)	
	distobuccal (DB)	I	59 (100.0%)	64 (100.0%)	-
	Palatal	I	58 (98.3%)	64 (100.0%)	0.296
		Mesiopalatal MP (I)	1 (1.7%)	0 (0.0%)	
	other configuration	Second mesiobuccal canal. MB2 (I)	7 (11.9%)	3 (4.7%)	0.193
		Disto-palatal DP (I)	1 (1.7%)	0 (0.0%)	
		NO	51 (86.4%)	61 (95.3%)	
	Number of Roots	3	51 (86.4%)	60 (93.8%)	0.172
		4	8 (13.6%)	4 (6.3%)	
	Number of Canals	3	37 (62.7%)	57 (89.1%)	0.001
		4	22 (37.3%)	7 (10.9%)	

**Table 5:** Association of prevalence of the morphology of the root canal types in mandibular first molars with respect to gender.

Variable			Male n (%)	Female n (%)	*p*-Value
Tooth #36 (first molar left side)	Mesio-buccal (MB)	I	59 (100.0%)	64 (100.0%)	-
	Mesiolingual (ML)	I	59 (100.0%)	63 (98.4%)	0.335
		II	0 (0.0%)	1 (1.6%)	
	Distal	I	41 (69.5%)	50 (78.1%)	0.449
		II	4(6.8%)	5 (7.8%)	
		III	1 (1.7%)	1 (1.6%)	
		IV	1 (1.7%)	2 (3.1%)	
		V	3 (5.1%)	0 (0.0%)	
		Distobuccal (I)	9 (15.3%)	6 (9.4%)	
	other configuration	Distolingual (I)	9 (15.3%)	6 (9.4%)	0.320
		No	50 (84.7%)	58 (90.6%)	
	Number of Roots	1	1 (1.7%)	0 (0.0%)	0.276
		2	0 (0.0%)	2 (3.1%)	
		3	49 (83.1%)	56 (87.5%)	
		4	9 (15.3%)	6 (9.4%)	
	Number of Canals	1	1 (1.7%)	0 (0.0%)	0.293
		2	0 (0.0%)	1 (1.6%)	
		3	40 (67.8%)	50 (78.1%)	
		4	18 (30.5%)	13 (20.3%)	
Tooth# 46 (first molar right side)	mesio-buccal (MB)	I	59 (100.0%)	64 (100.0%)	-
	mesiolingual (ML)	I	59 (100.0%)	63 (98.4%)	0.335
		II	0 (0.0%)	1 (1.6%)	
	Distal	I	40 (67.8%)	51 (79.7%)	0.178
		II	5 (8.5%)	2 (3.1%)	
		III	0 (0.0%)	2 (3.1%)	
		IV	2 (3.4%)	3 (4.7%)	
		V	2 (3.4%)	0 (0.0%)	
		Distobuccal (I)	10 (16.9%)	6 (9.4%)	
	other configuration	Distolingual (I)	10 (16.9%)	6 (9.4%)	0.212
		No	49 (83.1%)	58 (90.6%)	
	Number of Roots	2	1 (1.7%)	1 (1.6%)	0.456
		3	48 (81.4%)	57 (89.1%)	
		4	10 (16.9%)	6 (9.4%)	
	Number of Canals	2	1 (1.7%)		0.120
		3	39 (66.1%)	52 (81.3%)	
		4	19 (32.2%)	12 (18.8%)	

## Data Availability

The data presented in this study are available on request from the corresponding author. The data are not publicly available due to privacy and ethical concern.
